# Increase in submissions to International Brazilian Journal of Urology during Covid-19 quarentine

**DOI:** 10.1590/S1677-5538.IBJU.2020.05.01

**Published:** 2020-07-31

**Affiliations:** 1 Universidade do Estado de Rio de Janeiro - Uerj Unidade de Pesquisa Urogenital Rio de Janeiro RJ Brasil Unidade de Pesquisa Urogenital - Universidade do Estado de Rio de Janeiro - Uerj, Rio de Janeiro, RJ, Brasil; 2 Hospital Federal da Lagoa Serviço de Urologia Rio de Janeiro RJ Brasil Serviço de Urologia, Hospital Federal da Lagoa, Rio de Janeiro, RJ, Brasil

In times of Covid-19 quarentine we observed an increase in submissions around 38% to *International Brazilian Journal of Urology*, which is good news in these difficul times. The September-October number of Int Braz J Urol, the fifth under my supervision, presents original contributions with a lot of interesting papers in different fields: Prostate Cancer, Male Infertility, Female Incontinence, Renal Cell Carcinoma, Urinary Diversion, Hypospadia, Urinary Stones, Ureteral Cancer, myelomeningocele, and Testicular Cancer. The papers came from many different countries such as Brazil, USA, Turkey, China, Republic of Korea, Colombia, Spain and Canada, and as usual the editor's comment highlights some of them.

In the present issue we present three important papers about Prostate Cancer. Dr. Qin and colleagues from China performed in page 691 (1) a nice systematic review about the prostate cancer antigen 3 (PCA3) and suggested that PCA3 was a non-invasive method with the acceptable sensitivity and specificity in the diagnosis of Prostate cancer, to distinguish between patients and healthy individuals. Dr. Yingyi Qin and collegues from China presented in page 754 (2) a nice paper about perioperative outcomes between the robot- assisted radical prostatectomy (RARP) and open radical prostatectomy (ORP) and shows that the RARP approach has lower incidence rates of perioperative complications than the ORP approach, and there is a potential decreasing tendency of complication incidence rates for the RARP. The editor in chief would like to highlight the following works too:

Dr. Chavarriaga and collegues from Colombia (3) on page 743 evaluated the self-perception of health-related quality of life (HRQoL), ease of catheterization and global and cosmetic outcomes in patient's dependent on Mitrofanoff catheterization and concluded that continent urinary diversion is associated with good HRQoL, global satisfaction, ease and painless catheterization, adequate self-perception of cosmetic outcomes and a low complication rate, remaining a safe and viable option.

Dr. Ko and Collegues (4) from Republic of Korea perfomed on page 778 a interesting study about the association between preoperative retrograde pyelography (RGP), conducted to evaluate upper tract urothelial carcinoma (UTUC), and intravesical recurrence (IVR) after radical nephroureterectomy (RNU) and concluded that performance of RGP before RNU was shown to have a negative effect on IVR after surgery.

Dr. Gan and Collegues (5) from China performed on page 786 an interesting study about thea novel semirigid ureterorenoscope with irrigation and vacuum suction system and a modified ureteral access sheath (UAS) to overcome the deficiencies of the current procedure and to improve the efficiency and safety during treatment of upper urinary calculi. This study is on the cover in this number and concluded that this new ureterorenoscope is technically feasible, efficacious and safe for treatment of upper urinary calculi because of its advantages of high stone free rate and low complication rates.

Dr. Campos and Collegues (6) from Brazil and USA developed and validated on page 796 a new test of specific technical skills required for microsurgical varicocelectomy and suggested that the task-specific checklist of microsurgical varicocelectomy is reliable and valid in assessing microsurgical skills.

Dr. Amaral and Collegues (7) from Brazil analyzed on page 805 the serum and urinary markers of the Renin-Angiotensin-Aldosterone System (RAAS) in myelomeningocele patients with renal function abnormalities detected on DMSA and shows that the analysis of serum Angiotensin-Converting Enzyme (ACE), Angiotensin-Converting Enzyme 2 (ACE 2) and urinary ACE were not significant in patients with myelomeningocele and neurogenic bladder with renal injury previously detected by renal DMSA.

The Editor-in-chief expects everyone to enjoy reading and for sure better times will come soon.
